# A Comparison of Gender Differences in Smoking Behaviors, Intention to Quit, and Nicotine Dependence among Thai University Students

**DOI:** 10.1155/2018/8081670

**Published:** 2018-10-24

**Authors:** Dujrudee Chinwong, Ngamtip Mookmanee, Jongkonnee Chongpornchai, Surarong Chinwong

**Affiliations:** Department of Pharmaceutical Care, Faculty of Pharmacy, Chiang Mai University, Suthep Road, A. Muang, Chiang Mai 50200, Thailand

## Abstract

**Background:**

Smoking is the leading cause of preventable death. In Thailand, the prevalence of smoking is about 15-20 times higher among men than women. This study aimed to investigate gender differences among university students concerning smoking behaviors, nicotine dependence, and intention to quit smoking.

**Methods:**

A self-administered questionnaire was used to collect information from participants who were current smokers studying at a university in northern Thailand. Snowball sampling was used to recruit participants.

**Results:**

Of 364 participants, there were 321 males and 43 females. This study showed higher smoking behaviors among males than females; males were more likely to smoke every day than females (67.0 and 41.9%, respectively, p value=0.002), and the average number of cigarettes daily was higher among males than females (8.4 and 5.5, respectively, p value=0.006). The sources of cigarettes differed between males and females. The nicotine dependence level, as measured by the Fagerstrom Test for Nicotine Dependence, was quite low in both male and female smokers and did not differ significantly (mean score of 2.3±2.2 for males, 1.8±1.8 for females; p value=0.123). Females were more likely than males toward intention to quit in the next 30 days (51.2 and 34.0%, respectively, p value=0.041). The most common reason for intention to quit was awareness of harm to health, for which females were more concerned than males.

**Conclusion:**

Male and female university students who smoked differed in smoking behaviors and intention to quit, but not in nicotine dependence level. The university should provide health promotion to help students quit smoking.

## 1. Introduction

Smoking is the second leading risk factor for global disease burden, leading to more than six million deaths annually, worldwide [1]. In Thailand, tobacco use accounted for about one in ten of all deaths in 2009. The total economic loss due to smoking related diseases in Thailand was approximately 0.78% of GDP [2]. However, Thailand was among the earlier countries to introduce comprehensive restrictions on advertising and promotion concerning tobacco control leading to a range of activities for comprehensive tobacco control since then [3, 4].

Prevalence of smoking among males and females differs depending on the country. Developed and developing countries are similar in their mean adult male smoking prevalence (30.1 and 32%, respectively). On the contrary, developed countries have a much higher smoking prevalence among females than developing countries (17.2 vs. 3.1%) [5–7]. In general, smoking prevalence is higher among men than women, although prevalence varies among countries. In Thailand, the prevalence of smoking is about 15-20 times higher among men than women [8–10] depending on the survey. The Global Adult Tobacco Survey in 2009 and 2011 showed similar results of smoking prevalence, that is, about 46-47% for men and 2.6-3.0 for woman, and 24.0% for both men and women [10–12]. In Thailand, smoking prevalence has gradually declined over the past 2 decades, suggesting the tobacco control in Thailand using various strategies is effective [4, 13, 14]. However, smoking among youth seems to be rising. WHO's Global Adult Tobacco Survey showed the prevalence of smoking among Thai youths aged 15-24 years was at 19.8% in 2009 and increased to 21.7% in 2011 [11, 12].

Because many smokers start their first cigarette at university [7, 12, 15], university students should be monitored for their smoking behaviors to design tobacco prevention programs. According to smoking behavior surveys in Thailand, about 30% of current smokers started their smoking when they were studying at university [12, 15]. This is particularly true among females; one half of female university students initiated their smoking at the university [12].

In addition, smoke-free environments were established in Thailand regulated by the Non-Smokers' Health Protection Act 1992 (B.E. 2535) and the issued Ministerial Notification 19 prohibits smoking in public places, divided into 2 categories: Category one involves certain designated places that must be completely smoke-free where “smoking areas” are not allowed. Category 2 involves certain designated places that must be smoke-free but a “smoking area” may be specifically provided. The university is classified as category 2. That is, a university is a place that must be smoke-free; however, apart from buildings or structured areas, a “smoking area” may be specifically provided [16].

Smoking prevalence among Thai males and females differs widely; however, little is known about gender differences among university students who smoke regarding their smoking behaviors, intention to quit smoking, and nicotine dependence. Therefore, this study aimed to investigate gender differences in smoking behaviors, nicotine dependence, and intention to quit smoking among university students who smoke in northern Thailand. This will provide data for university committees to establish a smoke-free campus. In addition, policy makers can use the information about smoking behaviors, nicotine dependence, and intention to quit to monitor the smoking behaviors and promoting smoking cessation to the university students.

## 2. Materials and Methods

### 2.1. Study Design and Participants

This cross-sectional study was conducted in a university located in northern Thailand with about 28,000 students studying in 21 faculties including those related to medical and health sciences and social sciences as well as basic sciences and technology. Six faculties in the medical sciences comprised the Faculty of Medicine, Faculty of Dentistry, Faculty of Pharmacy, Faculty of Associated Medical Sciences, Faculty of Nursing, and Faculty of Veterinary Medicine. Ten faculties in the social sciences included the Faculty of Humanities, Faculty of Education, Faculty of Fine Arts, Faculty of Social Sciences, Faculty of Business Administration, Faculty of Economics, Faculty of Architecture, Faculty of Mass Communication, Faculty of Political Science and Public Administration, and Faculty of Law. Five faculties in basic science and technology consisted of the Faculty of Science, Faculty of Engineering, Faculty of Agro-Industry, Faculty of Agriculture, and College of Arts, Media and Technology.

Participants comprised undergraduate students (aged at least 18 years) studying at this university. The participants in this study were current smokers, defined as being a current smoker according to their self-report. Before asking the participants to voluntarily join this study, university students were assessed regarding their smoking status by answering the question, “Are you currently smoking cigarettes?”. When they said “yes,” they were classified as being a smoker and could participate in this study. When they said “no,” they could not participate.

The snowball sampling technique was used to recruit participants. That is, a smoker suggested their friends, who were smokers; then their friends were asked to voluntarily participate in this study. The students were explained about the study and asked to voluntarily complete the questionnaire which took about 10 minutes.

### 2.2. Data Collection and Questionnaire Development

A self-administered anonymous questionnaire was used to collect information from students. The questionnaire was created according to the objectives of the study and literature reviews to measure smoking behaviors, nicotine dependence, and intention to quit. The content validity of the questionnaire was examined by experts in smoking. Then the questionnaire was tested among 10 university student smokers for the use of appropriate language. This was also to ensure that the participants understood the written language in the questionnaire.

The questionnaires comprised 4 parts: sociodemographic data, smoking behaviors, nicotine dependence, and intention to quit smoking. First, the sociodemographic data consisted of sex, age, year of education, faculty of study, performance regarding education in terms of grade point average (GPA ranging from 0.00 to 4.00) and monthly expenditure (THB). Second, the smoking behaviors included frequency of smoking, amount of smoking in one day, and sources of cigarettes. Third, nicotine dependence was assessed using the Fagerstrom Test for Nicotine Dependence (FTND) consisting of 6 questions [17]. Questions with yes/no answers were scored as 0 or 1 and multiple choice questions were score from 0 to 3. Then the scores were summed, with higher score indicating higher dependence on nicotine. Scores from the FTND ranged from 0 to 10, dividing smokers into 3 groups: low nicotine dependence (score 0-3); moderate nicotine dependence (score 4-6); and high dependence (score 7-10). Fourth, the intention to quit smoking in this study was defined as the intention to quit smoking in the next 30 days. To be sure of students' quitting intentions, we confirmed their intention to quit or not using the questionnaire and interviewing. The students were first asked in the questionnaire whether they had the intention to quit in the next 30 days with reasons for their intention to quit as well as where to ask for help to quit smoking and their attempts to quit in the past. Then interviewing was used to confirm their answers regarding intention to quit in the next 30 days.

### 2.3. Statistical Analysis

STATA Software, Version 12 (StataCorp LP, College Station, TX, US), was used to statistically analyze the data with the significance level setting as two-tailed and at p value <0.05. Descriptive statistics for continuous variables were described as means ± standard deviation, while categorical data were reported as frequency and percentage. Differences between the two groups (males and females) were compared using Fisher's exact test for categorical variables, or independent t-test for continuous variables.

The sample size was calculated based on Yamane's formula for determining the sample size for a finite population with a margin of error set at 0.05 [18]. The prevalence of smoking among university students was 14.46%, reported by Luangla [19]. The number of students enrolled in the semester this study was conducted totaled 28,125 students, suggesting 4,067 were smokers. Thus, the sample size comprised 364 students. The sampling process was also stratified according to faculty.

### 2.4. Ethics Consideration

The study was conducted in accordance with the Declaration of Helsinki. The study protocol was approved by the Human Ethics Committee of the Faculty of Pharmacy, Chiang Mai University, before commencing (ethics approval number 28/2011; date of approval: 8 April 2011). All participants were informed about the study and all provided written informed consent.

## 3. Results

### 3.1. Participants' Characteristics

Of 364 university students, who completed the questionnaire, 321 (88%) were males and 43 (12%) were females. Mean age did not significantly differ: 21.2±1.5 years for males and 20.9±1.5 for females. Most university students were from faculties related to social sciences, sciences, and technology; students from faculties related to medical and health sciences totaled less than 10%. Their learning performance was mostly between 2.00 and 3.00, and their monthly expenditure was between 5,000 and 10,000 THB. Males and females differed regarding their learning performances, faculties, and expenditure (Table 1).

### 3.2. Smoking Behaviors

This study showed higher smoking behaviors among males than females; males were more likely than females to smoke everyday (67.0% and 41.9%, respectively, p value=0.002). Most students smoked less than 10 cigarettes daily. The average number of cigarettes daily was higher among males than females (8.4 and 5.5, respectively, p value=0.006). The sources of cigarettes differed between males and females. Males were more likely than females to buy cigarettes from a grocery store, while females were more likely than males to ask for cigarettes from friends (Table 2).

### 3.3. Nicotine Dependence

We employed the FTND to measure nicotine dependence. Most university students smoked less than 11 cigarettes daily after waking, and most smoke their first cigarette after 60 minutes. Most smoke more frequently during the rest of the day; the smoking time they hated most to give up was not the first in the morning. Most did not find it difficult not to smoke where it was forbidden, and most did not smoke when they were sick. This study showed males and females did not differ in nicotine dependence, and their mean scores of FTND were quite low, i.e., 2.3±2.2 for males and 1.8±1.8 for females (Table 3).

### 3.4. Intention to Quit, Reasons to Quit, and Places to Ask for Assistance

Our study showed that females were more likely than males toward intention to quit smoking in the next 30 days (51.2% and 34.0%, respectively, p value=0.041). One half of the respondents had at least one attempt to stop smoking. However, the past attempt to quit smoking did not differ between males and females. The most common reason for intention to quit was awareness of harm to health, for which females were more concerned than males. Places to ask for assistance to stop smoking were community pharmacies, hospitals, and Quitline 1600 (Table 4).

## 4. Discussion

To the best of our knowledge, this study was among the few studies to investigate gender differences concerning smoking behaviors of university students in Thailand. Although, in Thailand, males are 15 to 20 times more likely than females to smoke [8–10], some smoking behaviors differed. This study showed similarities and dissimilarities between male and female university students regarding their smoking behaviors, nicotine dependence, and intention to stop smoking.

### 4.1. Smoking Behaviors

This study showed that males were more likely than females to smoke every day, and the average number of cigarettes daily was higher among males than females. Tobacco smoking has declined during the past two decades in Thailand [8] as a result of many strategies for tobacco control in Thailand, such as advertising ban, health warnings on tobacco products, tax policy, and protection from tobacco smoke through regulations concerning no smoking areas in public and the work place [4, 8]. Despite a declining trend in smoking prevalence among males in Thailand [4, 11, 12], smoking prevalence remains higher among males than females. Possible reasons could be that some beliefs in smoking as well as social acceptability about smoking differ between males and females [3, 20]. Thai women are less likely to smoke, partly because smoking among females is considered undesirable in Thai society. Similar to related studies, females were more likely than males to believe that society disapproves of smoking [3, 20]. Thai males were more likely than females to believe that smoking makes young men look more attractive [3]. Males were more likely than females to believe that parents/guardians find smoking acceptable [3]. In addition, religious belief has an impact on smoking behaviors, attempts, and intention to quit [21, 22]. Most Thais are Buddhists, and they believed that their religion discourages smoking [22].

We found that university students could obtain cigarettes from various sources such as department stores, grocery stores, convenience stores, and asking for cigarettes from friends. The places to buy cigarettes differed between males and females. Males were more likely to buy cigarettes from a grocery store than females, while females were more likely than males to ask for cigarettes from friends. This may be due to the social unacceptability of female smoking; therefore, women will ask for a cigarette from a friend. Similarly, a study comparing Thailand and Malaysia found that about 20 to 30% of smokers received cigarettes from a friend [23]. Likewise, one study found that sources of substance use including tobacco were from peers [24].

According to the tobacco control law in Thailand, selling or providing any tobacco product to people aged less than 18 years is unlawful according to the Tobacco Products Control Act of A.D. 1992 [25]. In 2017, the legal age for people to buy cigarettes increased from 18 to 20 years according to the new law, the Tobacco Products Control Act of A.D. 2017 [26]. However, all participants in this study were aged at least 18 years old and they could legally buy cigarettes according to the law in 1992 which was in use during the time of conducting this study.

### 4.2. Nicotine Dependence

We used the FTND to measure nicotine dependence; that is, students smoked because of the addiction to nicotine in tobacco. This study showed no difference in nicotine dependence between male and female smokers. The mean low score (<3.0) of nicotine dependence among both males and females in our study suggested that male and female university smokers were addicted to nicotine at lower levels. In line with this study, both sexes had similar scores on the FTND, but males were more likely to smoke the first cigarette sooner after waking [27].

In addition to nicotine dependence, university students may smoke due to other reasons. Peer smoking, having family members smoking at home, and exposure to tobacco-related media were associated with smoking behaviors among adolescents [28]. Social anxiety was related to daily smoking especially among women [29]. Some students smoked occasionally; they used smoking mainly as a social engagement tool and to relieve negative emotions [30]. This was not in the scope of this paper; therefore, further research could investigate why university students smoked when they were not addicted to nicotine.

### 4.3. Intention to Quit, Reasons to Quit, and Places to Ask for Assistance

Our study showed that females were more likely than males toward their intention to quit smoking in the next 30 days. The top three reasons for quitting were influence from family members and friends, awareness of the dangers of smoking, and harm to health. Similar to a study by Branstetter et al. conducted among adolescent in the US, both sexes had equal perceptions that parents and family were supportive for a quit attempt [27]. However, women were more likely to have concerns regarding health than men. Our findings were in line with studies showing that females perceived the risk of dying from smoking significantly greater than males [29, 31, 32]. Our finding is similar to a study by Steptoe et al. that was conducted among university students from 23 countries showing that beliefs in the importance of not smoking for health were higher among females than males [7]. This was similar to related studies reporting that parents and peers were important individuals who could bring about smoking cessation among adolescents [28, 33].

One half of the respondents had at least one attempt to quit smoking in the past, but both males and females did not differ in their past attempts to quit smoking. Other studies also found that most smokers had tried to quit smoking and had made at least one attempt to quit [23, 34–36]. A study in Bangladesh by Hakim et al. found that intention to quit smoking was related to making an attempt to quit among adults [37]. Our study did not investigate the association between attempts and intention to quit, suggesting further research on this relationship is warranted.

Our findings showed that students seek help for quitting smoking from community pharmacies, hospitals, and Quitline 1600, but the response rate for this question was quite low, less than 15%. Other sources for seeking help in quitting smoking raised by students were students themselves, friends, family members, and relatives and the Internet. In Thailand, many places are available to provide assistance in quitting smoking, and mostly this service is free of charge. These places include community pharmacies, hospitals, and Quitline 1600. Our study suggested that students may not know where they could ask for assistance when they wanted to stop smoking. Quitline 1600 or the Thailand National Quitline is a telephone-based smoking cessation service, which has been provided in Thailand since January 2009 to help smokers to quit smoking. This service is quite convenient to use by calling 1600 [38]. However, the accessibility is still low, with less than 10% in this study having asked for help.

Smoking cessation services are available in some community pharmacies in Thailand where smokers can ask for help in quitting smoking [39, 40]. Two community pharmacies under the Faculty of Pharmacy of this university have provided smoking cessation services free of charge, but students may not know that this service is available in the university. The smoking cessation service at these community pharmacies should be promoted to students to encourage them to use the service to help them to quit smoking. In addition, the university should consider introducing a smoking cessation program for students if needed; this issue was also raised by the students in this study. Further research should be conducted to investigate how to promote smoking cessation services in the university and how to help students to quit smoking. A study conducted with students in India showed that antitobacco awareness programs have a potential in increasing the awareness of students concerning the dangers of smoking [41]. Thus, a study creating greater awareness of tobacco hazards among university students is warranted and the association of tobacco hazard awareness with their smoking behaviors, intention to quit, and smoking cessation could be investigated.

### 4.4. Implicaitons of the Findings

As Thailand has initiated the smoke-free university policy throughout the country in 2014, findings from this study should be useful to implement smoke-free campuses. The information regarding smoking behaviors, nicotine dependence, and intention to quit should be used to monitor tobacco use and support smoking cessation services for university students to encourage them to quit. Smoking cessation campaigns should be initiated and promoted for students that smoke especially those with intention to quit. The university should promote two community pharmacies under the Faculty of Pharmacy of the university to provide proactive smoking cessation services. The community pharmacies should be encouraged to reach target students who smoke.

### 4.5. Limitations

Some limitations should be noted. First, this study relied on self-reporting self-administered questionnaires. In addition, smoking is not quite acceptable in Thailand, particularly among women. Thus, respondents may have answered according to social norms in Thailand, especially females. However, self-reported smoking behaviors are a valid method to measure smoking consumption [42, 43]. Second, only a few participants came from faculties related to medical and health sciences: 26 (7%), 25 males and 1 female. No participants were from the Faculty of Nursing because this faculty mostly consists of women, even though we have endeavored to reach some participants from this faculty. These 26 participants may not represent smokers from medical and health sciences faculties. Third, representativeness should be concerned. This study was conducted at one university in northern Thailand using snowball sampling to reach participants because some people consider smoking undesirable in Thai society, especially female smoking. Obtaining access to smokers especially among university students remains difficult; therefore, the snowball sampling was used to reach participants. In addition, as the findings were from one university, representativeness is of concern. Therefore, the findings from this study may not be generalized to all university students in the country.

## 5. Conclusion

In conclusion, males and females differed in their smoking behaviors and intention to quit. Males were more likely to smoke every day than females; the average number of cigarettes daily was higher among males than females. Females were more likely than males toward intention to quit smoking in the next 30 days. However, their nicotine dependence levels did not differ and were quite low. This should motivate the university to provide smoking cessation services to students and health promotion for tobacco control in the university.

## Figures and Tables

**Table 1:** Participants' characteristics by sex (n=364).

Characteristic	Male (n=321)	Female (n=43)	p-value
Age (years)	21.2±1.5	20.9±1.5	0.160
Year			
1	8 (2.5)	4 (9.3)	0.238
2	67 (20.9)	9 (20.9)	
3	86 (26.8)	12 (27.9)	
4	99 (30.8)	10 (23.3)	
>4	61 (19.0)	8 (18.6)	
Faculties			
Medical and health sciences	25 (7.8)	1 (2.3)	0.002
Sciences and technology	158 (49.2)	11 (25.6)	
Social sciences	138 (43.0)	31 (72.1)	
Grade (learning performance out of 4.00)			
<2.00	30 (9.4)	1 (2.3)	0.004
2.00-3.00	234 (72.9)	25 (58.1)	
>3.00	57 (17.8)	17 (39.5)	
Expenditure monthly (THB)			
<5,000	69 (21.5)	3 (7.0)	0.020
5,000-10,000	209 (65.1)	37 (86.1)	
>10,000	43 (13.4)	3 (7.0)	

Note: numbers are n (%) or mean ± standard deviation (SD).

**Table 2:** Differences of smoking behaviors between males and females (n=364).

Smoking behaviors	Male (n=321)	Female (n=43)	p-value
Frequency of smoking			
Everyday	215 (67.0)	18 (41.9)	0.002
Not everyday	106 (33.0)	25 (58.1)	
Daily cigarette consumption			
1-5 cigarettes	149 (46.4)	26 (60.5)	0.065
6-10 cigarettes	96 (29.9)	13 (30.2)	
≥11 cigarettes	76 (23.7)	4 (9.3)	
Mean daily cigarette consumption	8.4±6.7	5.5 ±4.2	0.006
Sources of cigarettes			
Department store	25 (7.8)	3 (7.0)	1.000
Grocery store	145 (45.2)	11 (25.6)	0.021
Convenience store	216 (67.3)	23 (53.5)	0.087
Ask cigarettes from friends	95 (29.6)	22 (51.2)	0.008
Others provide cigarettes	50 (15.6)	12 (27.9)	0.052

Note: numbers are n (%) or mean ± standard deviation (SD).

**Table 3:** Differences of nicotine dependence as measured by the Fagerstrom Test for Nicotine Dependence (FTND) between males and females (n=364).

Nicotine dependence (FTND)	Male (n=321)	Female (n=43)	p-value
How many cigarettes a day do you smoke?			
10 or less (0)	245 (76.3)	39 (90.7)	0.224
11-20 (1)	69 (21.5)	4 (9.3)	
21-30 (2)	6 (1.9)	0 (0.0)	
31 or more (3)	1 (0.3)	0 (0.0)	
How soon after waking do you smoke your first cigarette?			
Within 5 minutes (3)	57 (17.8)	4 (9.3)	0.181
6-30 minutes (2)	64 (19.9)	7 (16.3)	
31-60 minutes (1)	45 (14.0)	11 (25.6)	
After 60 minutes (0)	155 (48.3)	21 (48.8)	
Do you smoke more frequently during the first hours after awakening than during the rest of the day?			
Yes (1)	58 (18.1)	5 (11.6)	0.392
No (0)	263 (81.9)	38 (88.4)	
Which cigarette would you hate most to give up?			
The first in the morning (1)	91 (28.4)	11 (25.6)	0.857
Any other (0)	230 (71.7)	32 (74.4)	
Do you find it difficult not to smoke in places where you should not, such as in a movie theater, at a library?			
Yes (1)	93 (29.0)	11 (25.6)	0.722
No (0)	228 (71.0)	32 (74.4)	
Do you smoke even if you are so ill that you are in bed most of the day?			
Yes (1)	81 (25.2)	9 (20.9)	0.707
No (0)	240 (74.8)	34 (79.1)	

Nicotine dependence*∗*			
Low (0-3)	230 (71.7)	38 (88.4)	0.071
Moderate (4-6)	72 (22.4)	4 (9.3)	
High (7-10)	19 (5.9)	1 (2.3)	
Mean score of FTND	2.3±2.2	1.8±1.8	0.123

Note: numbers are n (%) or mean ± standard deviation (SD); 0, 1, 2, and 3 indicating score for each answer; *∗*: score from 0 to 10 with higher score indicating the higher nicotine dependence.

**Table 4:** Differences of intention to quit smoking between males and females (n=364).

Intention to quit smoking	Male (n=321)	Female (n=43)	p-value
Intention to quit in the next 30 days			
Yes	109 (34.0)	22 (51.2)	0.041
No	212 (66.0)	21 (48.8)	
Attempt in quitting smoking			
Yes, >1 time	113 (35.2)	15 (34.9)	0.691
Yes, 1 time	46 (14.3)	4 (9.3)	
No	162 (50.5)	24 (55.8)	
Reasons for intention to quit			
Family members and friends	64 (19.9)	9 (20.9)	0.841
Social unacceptability	29 (9.0)	4 (9.3)	1.000
Inconvenience to smoke	17 (5.3)	4 (9.3)	0.292
Awareness of dangers of smoking	76 (23.7)	9 (20.9)	0.848
Health of smokers	65 (20.3)	15 (34.9)	0.048
Where to ask for help to quit			
Community pharmacy	23 (7.2)	5 (11.6)	0.354
Hospital	28 (8.7)	6 (13.9)	0.266
Quitline 1600	31 (9.7)	3 (7.0)	0.782
Others	72 (22.4)	10 (23.3)	0.848

## Data Availability

The data used to support the findings of this study are available from the corresponding author upon request.
